# Assessment of the Effects of Albendazole-Loaded Sulfonated Graphene Oxide on *Echinococcus granulosus* Protoscoleces: An *In Vitro* Investigation

**DOI:** 10.1155/2024/4851392

**Published:** 2024-09-27

**Authors:** Mohammad Reza Lashkarizadeh, Mohammad Shafie'ei, Mahdiyeh Lashkarizadeh, Seyed Mohammad Mousavi, Ghazaleh Sheibani, Zahra Akbari, Haniyeh Daneshafruz, Ali Derakhshani, Faham Khamesipour

**Affiliations:** ^1^ Research Center for Hydatid Disease in Iran Kerman University of Medical Sciences, Kerman, Iran; ^2^ Student Research Committee Faculty of Medicine Kerman University of Medical Sciences, Kerman, Iran; ^3^ Pathology and Stem Cell Research Center Department of Pathology School of Medicine Kerman University of Medical Sciences, Kerman, Iran; ^4^ Medical Student of First Faculty of Medicine Charles University, Kateřinskǎ 32, Prague 2 121 08, Czech Republic; ^5^ Faculty of Medicine Kerman University of Medical Sciences, Kerman, Iran; ^6^ Department of Chemistry Shahid Bahonar University of Kerman, Kerman 76169, Iran; ^7^ Halal Research Center of the Islamic Republic of Iran (IRI) Iran Food and Drug Administration Ministry of Health and Medical Education, Tehran, Iran

## Abstract

**Objectives:**

Due to Albendazole's relatively low efficacy and bioavailability, Echinococcosis has proven a challenge to manage successfully, with several studies investigating ways to improve the outcome, mainly showing mixed results. We, therefore, aimed to evaluate whether Sulfonated Graphene Oxide (S-GO), as nanocarriers, could improve the mentioned outcome.

**Methods:**

*Echinococcus* protoscoleces were divided into four groups based on the agent they received, which comprised control, S-GO, Albendazole, and Albendazole-loaded S-GO (S-GO-Albendazole). Then, the *Bax* and *Bcl-2* gene expression levels and the number of surviving protoscoleces in each group were determined.

**Results:**

*Bax* gene expression increased by 121% in the 50 *μ*g/ml concentration of the S-GO-Albendazole, while *Bcl-2* gene expression decreased by 64%. Moreover, S-GO-Albendazole was approximately 18% more effective at neutralizing protoscoleces than Albendazole and 14% and 31% more effective at improving the expression of the mentioned genes, respectively (*p* < 0.05). In addition, the number of surviving protoscoleces after exposure to the mentioned concentration reduced by approximately 99%.

**Conclusions:**

S-GO, despite not having significant lethality on protoscoleces, significantly increased the lethality of Albendazole and, therefore, is a suitable nanocarrier. However, we recommend conducting *in vivo* and clinical studies to more accurately determine this nanocomplex's potential and side effects.

## 1. Introduction

Echinococcosis, a zoonotic infection caused by the Taeniidea cestode family, has affected over 3 million individuals worldwide [1]. Moreover, the process of treating the disorder has not only been arduous but costly, too, amounting to more than 763 million dollars and 100,0006,62 disability-adjusted life years (DALYs) annually [2].

Besides, even though the mortality rate of Cystic Echinococcosis (CE) is estimated to be around 2–4%, the figure can go even higher if the infection is not adequately taken care of [1]. In this regard, several management techniques have been introduced during the past few decades, consisting of classic surgical removal, percutaneous sterilization or Puncture, Aspiration, Injection, and Re-aspiration (PAIR), plain follow-up of the cysts (the watch and wait method), and isolated medical therapy with anthelminthic agents [1, 3]. However, each technique has merits and shortcomings, leading to several newly introduced but not well-investigated methods [1, 3].

In addition, the conservative treatment, consisting of Albendazole (ABZ), which is a benzimidazole (BMZ), is only effective in a few inoperable cases (i.e., cases where multiple cysts have affected more than two organs), cases with peritoneal infiltrations, and before and after cyst operation (to prevent potential complications following spillages). Besides, their efficacy is thought to be overestimated, with recent studies showing full recovery in 18% to just over 50% of cases [4–7]. Furthermore, the current Albendazole (ABZ) formulations facing significant limitations. These include poor bioavailability, low water solubility, limited intestinal absorption (5%), and inadequate cyst-to-serum concentration ratios (25%). These challenges hinder the effective delivery of therapeutic ABZ concentrations to target tissues, compromising treatment efficacy. Consequently, there is an urgent need for innovative strategies to enhance the pharmaceutical properties and therapeutic performance of ABZ [1, 3–12].

Subsequently, numerous studies have been carried out to improve the mentioned low ABZ bioavailability, reporting various degrees of improvement. These methods include (a) Ingesting ABZ with fat-rich meals [13], (b) utilizing ABZ-containing sodium salts [14], (c) oil surfactant suspensions [15], and (d) incorporating ABZ or its active metabolite, ABZ sulfoxide (ABZ-SO), into nanoparticles or nanocapsules. Moreover, the most commonly utilized nanoparticles include metal nanoparticles (e.g., gold, zinc, copper, silver, and selenium), metal oxide nanoparticles (e.g., Zinc Oxide, Titanium Oxide, Cerium Oxide, and Zirconium Oxide), Chitosan and its derivatives, Liposomes, Solid lipid nanocapsules (SLN), ABZ-poly-lactic-co-glycolic-acid (ABZ-PLGA) nanoparticles, and Poloxamer 188 [11, 16–23]. Nevertheless, there is still much to be known, and some believe that other not-yet-utilized nanoparticles can achieve even higher feats.

One such group is the Graphene family of nanoparticles (GFN), a carbon-based, up-and-coming, yet cost-effective family of nanomaterials with relatively low environmental hazards [24, 25]. There are several members in the family, with the most notable being Graphene Oxide (GO), Reduced Graphene Oxide (rGO), Graphene quantum dots (GQD), and Graphene Nanoribbons (GNR) [25]. In addition, their characteristics, including their sizable surface area, high loading capacity, highly desirable thermal and mechanical properties, pH responsiveness, and capable enhanced permeability and retention (EPR) effects, have been well described in the literature [25–27]. Their toxic effects have also been investigated on *C. elegans* worms and *Zebrafish*, but the results were not as straightforward, with significant toxicity shown to the former but no adverse effects on the latter [28, 29]. In addition, their effects on human cells are not yet fully comprehended, with studies reporting negligible to significant toxic effects based on the utilized nanocomponent's size, coating, and structure [26].

Nevertheless, the positive effects of GFNs have been widely studied, which include their interesting potential in drug and gene delivery systems, tissue engineering and regeneration, and antimicrobial regimens [30–34]. For instance, the usefulness of the GFN compounds in drug delivery has been demonstrated on anticancer agents Rituxan and Doxorubicin (DOX) and antibiotics such as gentamicin, ciprofloxacin, and benzylpenicillin [35–40]. However, anthelminthic agents have still not been the subject of such studies. Therefore, based on the promise this agent could provide, we evaluated the effects of the graphene-based nanocomposites (namely, sulfonated GO or S-GO) on the efficacy of ABZ on Echinococcus protoscoleces (PSCs).

## 2. Materials and Methods

### 2.1. Design

**Figure 1:**
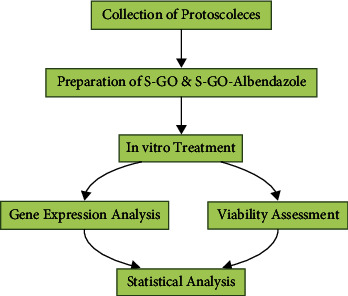
Illustration of the study design.

The present *in vitro* investigation was conducted on viable protoscoleces (PSCs) of the sheep strain of *E. granulosus*, subsequent to obtaining protocol approval from the local Institutional Review Board (IRB). Additionally, efforts were made to minimize the number of animals utilized, ensuring only the necessary quantity to achieve the required PSC counts. An illustration of the study design is provided below. (see Figure 1).

### 2.2. PSC Collection

We collected the livers and lungs of the infected sheep (kept under appropriate and ethically approved minimally harmful conditions) from a local industrial slaughterhouse and transferred them to the parasitology laboratory under sterile and thermally isolated conditions. After disinfecting the cysts' surfaces with 70% ethyl alcohol, 10–50 ml of the cyst fluid was aspirated using a sterile syringe and transferred to a 50 ml falcon tube, while the germinal layer was also isolated and added to the tube with the mix left to rest for 5 minutes. Then, the supernatant fluid was removed, and the obtained PSCs, which settled down at the bottom, were washed five times with a mixture of Phosphate-Buffer Saline (PBS, pH = 7.1) and Penicillin-Streptomycin (Biosera, USA). Then, samples with >90% viability (determined via the 0.1% eosin stain produced by dissolving 1 g of eosin powder in 1000 mL of distilled water) were deemed suitable [41]. These PSCs (5000 PSCs per 100 microliter of fluid) were then cultured in a medium comprising 10 ml of RPMI 1640, 10% Fetal Bovine Serum (FBS; Gibco, Germany), and 1% Penicillin-Streptomycin in aseptic conditions [42–45].

### 2.3. Preparation of GO

GO was synthesized using the modified Hummer's method and the graphite powder as raw material [46]. First, 2 g of the powder and 1.5 g of sodium nitrate were slowly added to a flask containing 70 ml of concentrated sulfuric acid (H2SO4), which was placed in an ice bath, and the mixture was stirred at a temperature of 0–5°C. After homogenization, 8 g of potassium permanganate (KMnO_4_) was added gradually for 2 hours. Then, the temperature of the reaction mixture was increased to 35°C and stirred for one hour to obtain a light brown viscose mixture. Next, 100 ml of distilled water was slowly added while stirring the solution, with the temperature increased to 90°C for one hour. The reaction was then stopped by adding 20 ml of 30% hydrogen peroxide (H_2_O_2_), and the mixture was stirred for 24 hours at room temperature. Then, the resulting suspension was washed with 3% hydrochloric acid (HCl) and distilled water until a pH of seven was achieved. Finally, the neutralized solution was sonicated using an ultrasonic homogenizer with an output power of 40% for two hours. The resulting brown solution was centrifuged to remove unlaminated graphite oxide sheets. The final step was drying the graphene oxide solution using a freeze-dryer to obtain graphene oxide powder with laminated plates.

### 2.4. Preparation of S-GO

To synthesize S-GO, 1 g of GO was added to a flask containing 350 mL of distilled water and then ultrasonicated for 30 minutes. Afterward, 5 g of sodium nitrite (NaNO_2_), dissolved in 75 ml of distilled water, was added to the mix and placed in an ice water bath. Additionally, 4 g of sulfanilic acid (C_6_H_7_NO_3_S) was dissolved in 50 ml of HCl (1 M) and transferred to a burette. In the next step, to synthesize S-GO through diazonium salt formation, the solution was drop-by-drop added to the flask containing GO and NaNO_2_ (within a temperature of 0 to 5°C) and stirred for 24 hours. Then, the resulting mixture was washed, centrifuged with distilled water to reach a neutral pH, and dried by a freeze-dryer.

### 2.5. Synthesis of ABZ-Loaded S-GO Nanocomposite

For this purpose, 5 mg ABZ and 2.5 mg S-GO (2 : 1 ratio) were added to a flask to synthesize a 50 *μ*g/ml ABZ-loaded S-GO (S-GO-ABZ) solution, with distilled water added until the solution reached 100 ml. The solution was then exposed to ultrasonic waves for 30 minutes to disperse completely. Finally, the ABZ-loaded S-GO solution was stored in the refrigerator at 5°C and in dark conditions. Furthermore, other concentrations were prepared similarly, with an ABZ-to-S-GO ratio of 2 : 1.

### 2.6. Nanocomposite Characterization

Fourier-transform infrared spectroscopy (FT-IR) was used to identify functional groups on the S-GO samples. X-ray diffraction (XRD) analysis using a Panalytica X'Pert PRO powder diffractometer with Cu K*α* radiation (*λ* = 1.5404 Å) was employed to study the crystallinity of the materials.

### 2.7. RNA Isolation and Complementary DNA (cDNA) Synthesis

After 60 min, we isolated the cellular RNA from the study groups on the desired timescales using an RNX-plus kit (CinnaGen, Iran) and determined the quantity and quality using the NanoDrop 2000c spectrophotometer (Thermo Scientific, USA). The resulting RNA was then reverse-transcribed into cDNA using a reverse transcriptase kit (Thermo Scientific, USA) and taken into account as the PCR amplification template.

### 2.8. Primer Design and Quantitative Real-Time Polymerase Chain Reaction (qRT-PCR)

We considered B-cell lymphoma 2 (Bcl-2) and BCL2 Associated X, Apoptosis Regulator (Bax) genes as the determinants of PSC apoptosis. Initially, we checked the Deoxyribonucleic Acid (DNA) and Ribonucleic acid (RNA) sequences of the genes on the genome databases of the National Center for Biotechnology Information (NCBI; available at https://www.ncbi.nlm.nih.gov) and European Bioinformatics Institute (Ensembl; available at https://www.ensembl.org). Then, after identifying different exons of each gene, we designed the appropriate primers (Isogen, Netherlands), provided in Table 1, using the Primer3 tool (available at https://frodo.wi.mit.edu/cgibin/primer3/primer3_www.cgi), while also double checking their integrity using the 3.5^th^ edition of the Gene Runner tool (Hasting Software, Inc., 1994). We then assessed the specificity of the primers via the Basic Local Alignment Search Tool (BLAST).

Ultimately, we determined the expression levels of Bcl-2 and Bax (at baseline and four weeks after cell differentiation) via performing the qRT-PCR in 15 *µ*l reaction volume containing 1 *μ*l of cDNA, 4.5 *μ*l of sterile deionized water, 7.5 *μ*l of FastStart SYBR Green Master ROX solution (Roche), and 2 *μ*l of the mentioned primers in the FAM/SYBR and ROX channels of the ROTOR GENE 3000 (Corbett) device. The resulting data from the former channel were normalized with the latter channel and transferred to the LinRegPCR tool (available at https://www.gene-quantification.de/). These data from the band intensities were then normalized against their primary internal control gene concentrations to determine their expression levels objectively.

### 2.9. *In Vitro* Treatment and Scolicidal Assay

To minimize bias in allocating treatment, PSCs were randomly divided into four main groups based on the agent and dosage they received: (1) negative untreated control, (2) ABZ, (3) S-GO, and (4) S-GO-ABZ. These groups also comprised three subgroups with 10 *µ*g/ml, 20 *µ*g/ml, and 50 *µ*g/ml concentrations, respectively. Finally, the agents' scolicidal effects were assessed after 15, 30, and 60 minutes. Blinding was used when assessing the results of PSC viability and gene expression to further reduce potential bias.

### 2.10. Statistical Analysis

We used the 27^th^ version of SPSS statistical software (IBM Corp. Released 2020. IBM SPSS Statistics for Windows, Version 27.0. Armonk, NY: IBM Corp) to analyze the obtained data. Moreover, the differences between the means of the PSC viability in the active and control groups were analyzed using the One-way analysis of variance (ANOVA) with the Student–Newman–Keuls (SNK) method as the post hoc analysis. Moreover, *p* < 0.05 were considered statistically significant.

## 3. Results

### 3.1. FT-IR Spectra

**Figure 2:**
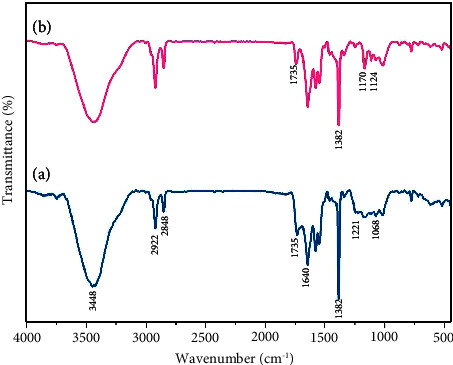
FT-IR spectra of GO (a) and S-GO (b).

In Figure 2, we compared the spectra of (a) GO and (b) S-GO. In Figure 2(a), the broad peak at 3448 cm^−1^ is related to the O–H stretching vibration caused by functional hydroxyl and carboxyl groups. Moreover, the peaks that appeared at 2922 and 2848 cm^−1^ are assigned to the C-H stretching vibrations, while the strong peak at 1382 cm^−1^ is assigned to the C-H bending vibrations. The peak at 1735 cm^−1^ is also attributed to the stretching vibration of the carbonyl group (C=O), while the peak at 1640 cm^−1^ is attributed to the stretching vibration of aromatic rings (C=C). The 1221 and 1068 cm^−1^ peaks also correspond to OC (due to C-O-C) and OC (due to C-OH of alcohol groups) stretching vibrations, respectively. Finally, according to the peaks showing oxygen presence, the graphite oxidation reaction can be confirmed [47].

The peaks corresponding to S-GO are shown in Figure 2(b). The peaks at 1170 and 1124 cm^−1^ are related to the stretching vibrations of two S-O groups and confirm the presence of sulfonic acid. It is also observed that after the sulfonation process, the peaks of 1735 and 1382 cm^−1^ have weakened [48, 49] (Figure 2).

### 3.2. XRD Pattern of S-GO

**Figure 3:**
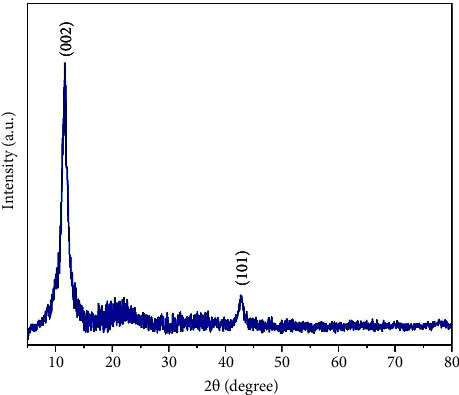
XRD patterns of S-GO.

The pattern was recorded for the GO sample in the range of 5° to 90° (2*θ*) at room temperature, at a temperature increase speed of 0.02°/s is depicted in Figure 3. The structural information about GO shows two characteristic peaks at 11.5° and 42.8° angles due to the reflections of (002) and (101) planes [50] (Figure 3).

#### 3.2.1. Gene Expression

In the S-GO group, the Bax gene expression increased by 12% in the 10 *μ*g/dl concentration, 14% in the 20 *μ*g/dl concentration, and 32% in the 50 *μ*g/dl concentration, respectively. Moreover, the expression of the Bcl-2 gene decreased by 7%, 9%, and 13% in the mentioned concentrations, respectively.

In the ABZ group, the Bax gene expression increased by 10%, 30%, and 90% in the 10 *μ*g/dl, 20 *μ*g/dl, and 50 *μ*g/dl concentrations, respectively, while Bcl-2 expression decreased by 9%, 18%, and 49% in the mentioned concentrations.

**Figure 4:**
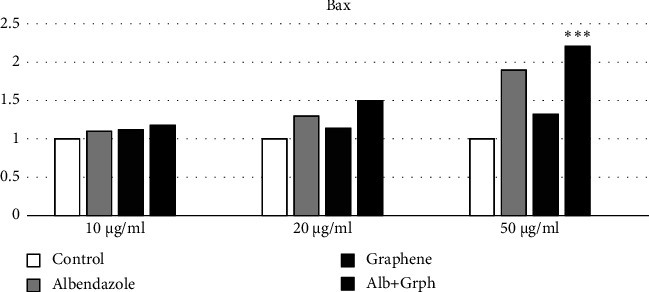
Bax gene expression in the S-GO-ABZ, ABZ, S-GO, and control groups. However, only the increase in the S-GO-ABZ group was significant. Bars represent mean ± standard deviation (SD) from duplicate experiments. (^∗^*P* < 0.05, ^∗∗^*P* < 0.01, ^∗∗∗^*P* < 0.001) (Alb, Albendazole; Grph, S-GO).

**Figure 5:**
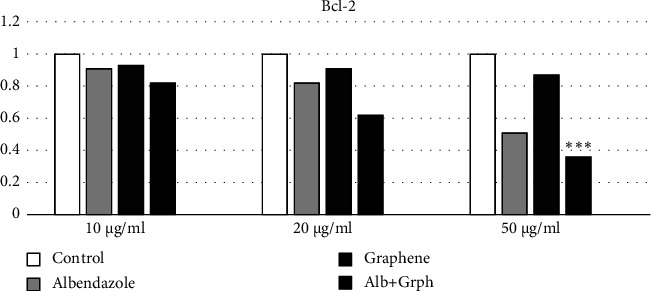
Bcl-2 gene expression in the S-GO-ABZ, ABZ, S-GO, and control groups. However, only the decrease in the GFN-ABZ was significant. (^∗∗∗^*p* < 0.05) (Alb, Albendazole; Grph, S-GO).

In the S-GO-ABZ group, the Bax gene expression increased by 18%, 50%, and 121% in the 10 *μ*g/dl, 20 *μ*g/dl, and 50 *μ*g/dl concentrations, respectively, while Bcl-2 expression decreased by 18%, 38%, and 64%, respectively. The analyses showed, however, that only the changes in the S-GO-ABZ group's 50 *μ*g/ml concentration were statistically significant (*p* < 0.05) (Figures 4 and 5).

### 3.3. PSC Viability

**Figure 6:**
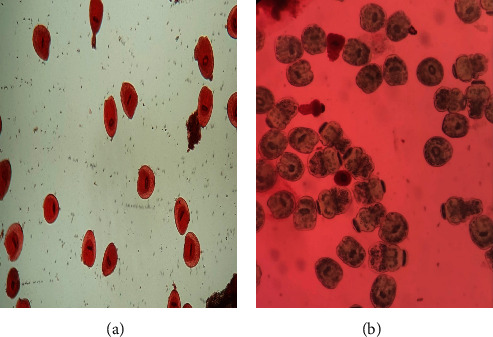
(a) The dead and (b) viable protoscoleces (PSCs) after treatment with S-GO-ABZ.

As shown in Figure 5(a), dead PSCs were stained red after exposure to eosin, while in Figure 5(b), viable ones were unstained with flame cell activity. Moreover, the effects of various agents (i.e., ABZ, S-GO, and S-GO-ABZ) on PSCs at different intervals and concentrations are shown in Figure 6.

**Figure 7:**
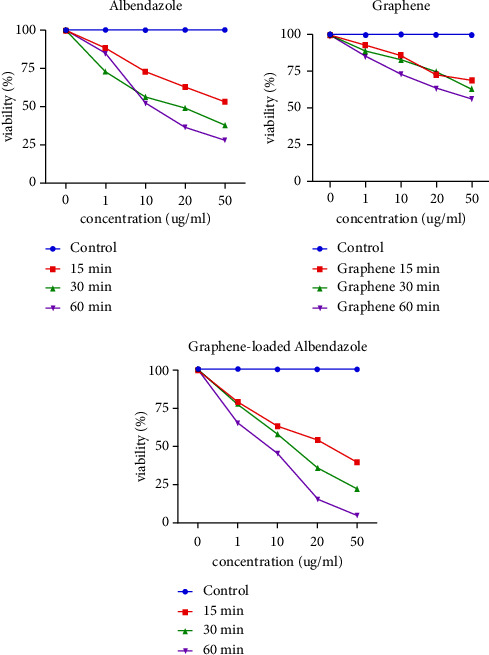
Number of viable protoscoleces (PSCs) after applying different concentrations of each agent in the S-GO-ABZ, ABZ, S-GO, and control groups. However, only the decreases in the S-GO-ABZ and ABZ groups were significant (*p* < 0.05).

According to the observed results, the number of viable PSCs decreased by 2% when the 1 *μ*g/ml concentration of S-GO was applied, by 7% when the 10 *μ*g/ml concentration was applied, by 14% when the 20 *μ*g/ml concentration was applied, and by 18% when the 50 *μ*g/ml concentration was applied. Furthermore, following the application of the mentioned concentrations, the decreases in the PSC counts of the ABZ group were 3%, 34%, 69%, and 81%, respectively. In the S-GO-ABZ group, the mentioned decreases were 24%, 49%, 93%, and 99%, respectively. However, such decreases were more significantly pronounced in the S-GO-ABZ group compared to the ABZ group (*p* < 0.05) (Figures 6 and 7).

## 4. Discussion

One of the reasons for the difficulty in controlling and managing Echinococcosis is the low effectiveness of Albendazole, for which no suitable replacement has yet been discovered. However, the findings of our study indicate that at a specific concentration (50 *μ*g/ml), S-GO-ABZ is approximately 18% more effective than ABZ in neutralizing protoscoleces and 14% and 31% more effective in altering the expression of Bax and Bcl-2 apoptosis regulator genes, respectively (i.e., increase in Bax expression and decrease in Bcl-2 expression).

However, based on our literature review, this study was the first to investigate the effectiveness of S-GO as a nanocarrier on the toxicity of ABZ on Echinococcus PSCs. Therefore, we could only attempt to compare our findings with previous remotely similar studies to achieve a relative *in vitro* efficacy profile. One study investigated the combined effects of gold nanoparticles incorporation and lasers on Echinococcus PSCs, with an 89.3% toxicity rate after 120 minutes [51]. In another study, the PSC toxicity of copper, iron, silver, silicon, and zinc nanoparticles was investigated after 60 minutes of exposure, demonstrating that zinc nanoparticles possessed the highest toxicity (80%), followed by silicon (52%) [52]. Another similar study investigated the alterations in caspase-3 gene expressions and DNA fragmentation rates after exposure to ABZ-PLGA, which, even though ABZ and ABZ-PLGA both demonstrated significant toxicities, the differences between them were not statistically significant [53].

Moreover, another study assessed the PSC toxic effects of ABZ-loaded copper nanoparticles (with a concentration of 750 mg/ml) along with alterations in caspase-3 gene expression, demonstrating that even though ABZ-loaded copper nanoparticles neutralized 100% of the PSCs after 60 minutes and decreased the caspase-3 expression by 36%, the nanoparticle alone killed 73%, therefore, not showing how copper nanoparticles improved ABZ drug delivery and obscuring the results. Nevertheless, even though our study achieved a similar toxicity profile, the PSC toxicity of S-GO alone was negligible, objectively showing its capabilities in improving the effectiveness of ABZ purely as a nanocarrier.

Ultimately, we acknowledge that the investigation of the nanocarrier profile of GFNs for ABZ is in its first steps, and several steps need to be taken for them to be considered. Therefore, this *in vitro* study may not completely mimic the intricate *in vivo* surroundings. Further *in vivo* studies are needed to confirm the effectiveness and safety of S-GO-ABZ in animal models before it can be used clinically, as the interactions among the nanocomposite, the host immune system, and the parasite in the hydatid cyst may impact treatment results. Furthermore, the research did not examine the extended-term durability and distribution timing of Albendazole from the S-GO nanocarrier. Future research should determine the drug release characteristics of S-GO-ABZ and improve the formulation for extended drug release.

Additionally, while our study focused on protoscolex viability as a measure of antiparasitic activity, future research could explore other aspects of *E. granulosus* biology, such as the effects of ALB-GO and S-GO on cyst development, parasite migration, and host immune responses. Understanding the broader impact of these nanomaterials on the parasite life cycle could provide valuable insights into their therapeutic potential.

## 5. Conclusions

Despite not having significant toxicity on Echonicoccus PSCs, S-GO substantially increased ABZ's toxicity. Therefore, we believe that S-GO can be considered a suitable nanocarrier for improving the therapeutic efficacy of ABZ in Echinococcosis. By elucidating their mechanisms of action and addressing current limitations, further research can significantly advance the development of effective and sustainable therapies for this neglected tropical disease.

## Figures and Tables

**Table 1:** The sequences of the designed primers.

Gene	Primer sequence
Bcl-2	F: AAGCATCAACCAATCTGTCAGAG R: ACTATCGAAGGAGGCATAAATGTC

Bax	F: TCCTCCTCGGATGAAGCAGA R: CTGATGTACCGGTCTCTCGC

5.8S	F: GTCGATGAAGAGTGCAGCCAAC

## Data Availability

The dataset supporting the conclusions of this manuscript is available from the corresponding author upon reasonable request.
